# Efficacy of syringe-irrigation topical therapy and the influence of the middle turbinate in sinus penetration of solutions^☆^

**DOI:** 10.1016/j.bjorl.2016.06.013

**Published:** 2016-07-31

**Authors:** Guilherme Henrique Wawginiak, Leonardo Balsalobre, Eduardo Macoto Kosugi, João Paulo Mangussi-Gomes, Raul Ernesto Samaniego, Aldo Cassol Stamm

**Affiliations:** a Complexo Hospitalar Edmundo Vasconcelos, Centro de Otorrinolaringologia e Fonoaudiologia, São Paulo, SP, Brazil; b Universidade Federal de São Paulo, Escola Paulista de Medicina, Departamento de Otorrinolaringologia e Cirurgia de Cabeça e Pescoço, São Paulo, SP, Brazil

**Keywords:** Sinusitis/therapy, Therapeutic irrigation, Video-assisted surgery, Natural orifice endoscopic surgery, Sinusite/terapia, Irrigação terapêutica, Cirurgia vídeoassistida, Cirurgia endoscópica por orifício natural

## Abstract

**Introduction:**

Topical therapies are the best postoperative treatment option for chronic rhinosinusitis, especially those with high volume and pressure, such as the squeeze bottles. However, they are not an available option in Brazil, where irrigation syringes are used.

**Objective:**

To investigate the efficacy of topical sinonasal therapy with syringe and the influence of the middle turbinate on this process

**Methods:**

Intervention study in training models (S.I.M.O.N.T.). After standard dissection, three interventions were performed (Nasal Spray 4 puffs, 60-mL syringe and 240-mL Squeeze Bottle) with normal and Sutured Middle Turbinate. Images of each sinus were captured after the interventions, totalizing 144 images. The images were classified by 10 evaluators according to the amount of residual volume from zero to 3, with zero and 1 being considered poor penetration and 2 and 3, good penetration. The 1440 evaluations were used in this study.

**Results:**

Considering all middle turbinate situations, the amount of good penetrations were 8.1% for Spray; 68.3% for Syringe, and 78.3% for Squeeze (*p* < 0.0001). Considering all types of interventions, the Normal Middle Turbinate group had 48.2% of good penetrations and the Sutured Middle Turbinate, 55% (*p* = 0.01). Considering only the Sutured Middle Turbinates, there was no difference between the interventions with Syringe and Squeeze (76.3% *vs.* 80.4%; *p* = 0.27).

**Conclusion:**

Topical therapy of irrigation with a 60-mL syringe was more effective than that with nasal spray. The status of the middle turbinate proved to be fundamental and influenced topical therapy. Irrigation with syringe was as effective as the squeeze bottle when the middle turbinate was sutured to the nasal septum.

## Resumo

**Introdução:**

Terapias tópicas são a melhor opção de tratamento pós-operatório da rinossinusite crônica, principalmente com alto volume e pressão, como os *squeeze bottles*. Porém, não são opções disponíveis na realidade brasileira, onde frequentemente são usados seringas para a irrigação.

**Objetivo:**

Averiguar a eficácia da terapia tópica nasossinusal com seringa e a influência da concha média neste processo.

**Método:**

Estudo de intervenção em modelos de treinamento (S.I.M.O.N.T.). Após dissecção padronizada, três intervenções foram realizadas (spray nasal 4 *puffs*, seringa de 60 mL e *squeeze bottle* de 240 mL) com a concha média normal e suturada. Foram capturadas imagens de cada seio após as intervenções, totalizando 144 imagens. As imagens foram classificadas por 10 avaliadores de acordo com a quantidade de volume residual de zero a 3, sendo zero e 1 considerados penetração ruim e 2 e 3, penetração boa. As 1.440 avaliações foram utilizadas neste estudo.

**Resultados:**

Considerando todas as situações de concha média, a quantidade de penetrações boas foram 8,1% para spray; 68,3% para seringa e 78,3% para *squeeze* (*p* < 0,0001). Considerando todos os tipos de intervenção, a concha média normal obteve 48,2% de penetrações boas e a concha média suturada, 55% (*p* = 0,01). Considerando apenas concha média suturada, não houve diferença entre as intervenções seringa e *squeeze* (76,3% *vs*. 80,4%; *p* = 0,27).

**Conclusão:**

A terapia tópica de irrigação com seringa de 60 mL foi mais eficaz que com spray nasal. O status da concha média mostrou-se fundamental e influenciou a terapia tópica. A irrigação com seringa foi tão eficaz quanto à com *squeeze bottle* quando a concha média foi suturada ao septo nasal.

## Introduction

Chronic Rhinosinusitis (CRS) is defined as a chronic inflammatory process of the nasal mucosa and paranasal sinuses, lasting more than 12 weeks without complete resolution of symptoms.^1^, ^2^ This chronic inflammation of the mucosa can be caused by several distinct pathophysiological mechanisms, making the term CSR an “umbrella” term that houses several different diseases presenting with nasal obstruction, rhinorrhea, olfactory changes and/or facial pain.^3^

Nasal irrigation, despite the growing interest in recent years^4^, ^5^ is a very old treatment that has been practiced for centuries in India during the practice of yoga, and since the 19th century in Western medicine.^6^ Historically, it has employed several solutions, such as sodium chloride, and different instruments, such as syringes and bottles for nasal washings.^7^ Although most of these agents are not currently used, the practice of nasal irrigation with saline solution persisted, and has gained popularity throughout the 20th century. It is now considered effective in the control of sinonasal disease and is a cornerstone in the postoperative care.^8^

Several nasal irrigation techniques and devices have been developed over the years, with variations in the applied solution pressure and volume. Low-volume devices utilize around 100 μL in sprays up to a few milliliters for droppers, atomizers and nebulizers. The high-volume systems deliver from 50 mL to 240 mL through squeeze bottles and neti pots. Following Endoscopic Sinonasal Surgery (ESS), the high-volume irrigation systems provide good distribution of the solution in the nasal cavity and cribiform plate, and particularly have better penetration into the sinus. The greater the volume of irrigation, the greater the distribution to the sinus cavities.^9^, ^10^, ^11^

Therefore, ESS primary role in CSR is now to prepare the paranasal sinuses to receive topical medications, especially in the form of irrigation.^12^ Technical factors may impair proper distribution of irrigation, such as synechiae, especially between the Middle Turbinate (MT) and the lateral nasal wall.^13^ Frequently the middle turbinate is mobilized and, not infrequently becomes instable during surgery, leading to its lateralization and/or adherence.^13^, ^14^, ^15^

The controversy between preserving or removing the MT is as old as the history of ESS. Those who condemn MT resection speak of the change of nasal function, the risk of frontal CRS, the loss of an important anatomical landmark for revision surgeries, the risk of anosmia, or the excessive formation of scar tissue. Those who favor partial or total resection of MT believe in the benefits in enhanced postoperative care, reduction of synechiae, and greater accessibility to the sinuses.^16^, ^17^, ^18^ An intermediate approach to these two views include the use of spacer devices in the middle meatus and suturing the middle turbinates to the nasal septum.^14^, ^15^

In Brazil, there are no marketed devices that provide high solution volumes with pressure, so the local reality is the use of syringes for the administration of corticosteroids solutions.^19^ However, there is no evidence that this type of application is similar to techniques already described, nor is there evidence of the influence of the middle turbinate in this topical therapy modality. Therefore, the aim of this study was to examine the efficacy of topical nasosinusal therapy with a syringe, and the influence of the middle nasal concha in this process.

## Methods

This is an intervention study in a model of sinonasal dissection, where three different interventions of topical therapy were tested in two specific groups of position of the middle turbinate.

**Figure 1:**
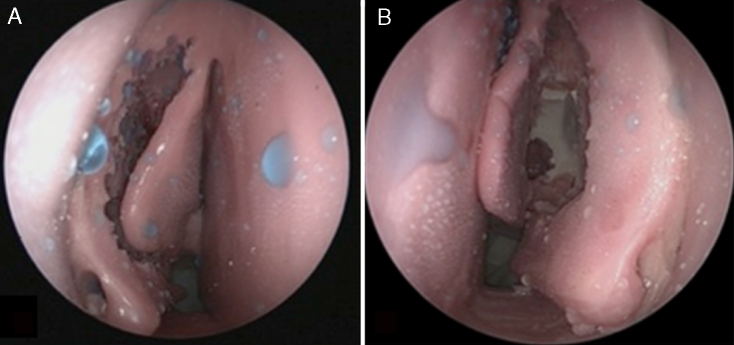
Model after standard dissection. (A) Middle turbinate kept with no treatment after dissection. (B) Middle turbinate sutured to the septum after dissection.

We used three units of the sinus model otorhino-neuro training of endoscopic surgery (SIMONT) produced by Pro Delphos^®^ (Recife, Brazil), in which the same otolaryngologist surgeon (LLBF) conducted standardized surgical dissection (fronto-spheno-maxillo-ethmoidectomy) in both nasal cavities, totaling six nasal cavities dissected. All three models have received, in addition to standard surgical dissection, two sequential treatments of the middle turbinate, corresponding to the division into two groups: first, the middle turbinate was kept in its usual position after dissection and matched the Normal Middle Turbinate group (Fig. 1A). Secondly, the middle turbinates were sutured together with a single stitch of Nylon 3–0 suture transfixing the nasal septum (Fig. 1B), with the group being called Sutured Middle Turbinate group.

**Figure 2:**
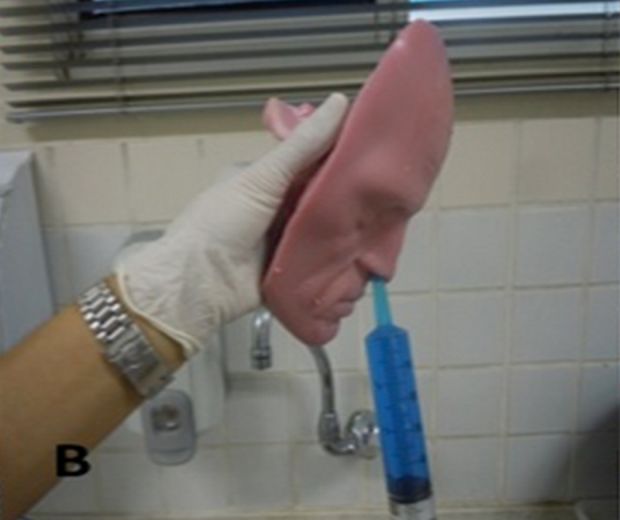
Topical therapy of irrigation with a syringe, performed in orthostatic position, simulating slight anterior head flexion (30°).

In each model, three interventions of topical therapy were performed. The lavage solution was prepared by diluting 10 drops of blue food dye in 500 mL of water. A single investigator performed all interventions of topical therapy with the S.I.M.O.N.T. model in orthostatic position, simulating anterior flexion of the neck of about 30° (Fig. 2). The first intervention consisted of four atomizations of nasal spray in each nostril, which corresponded to 0.2 mL of solution per nostril, and it was called Spray intervention. In the second intervention, a 60-mL syringe with catheter tip (Injex, Ourinhos, SP, Brazil) was used, with all this volume injected into a single application under pressure in each nostril, and this intervention was called Syringe intervention. And the last intervention was performed with a 240-mL bottle (squeeze bottle) for high-volume, high-pressure topical therapy (Sinus Rinse, NeilMed Pharmaceuticals, Inc., Santa Rosa, California, EUA), with half of this volume injected into a single application under pressure in each nostril, and was called Squeeze intervention.

After each intervention, a 30° rigid endoscope connected to a recording system with a camera and light source was introduced in the nasal fossae, with endoscopic images of the maxillary, ethmoid, frontal and sphenoid sinuses being captured, always taking care not to allow the identification of the treatment performed in the middle turbinate. After registration, the model was washed with running water and suctioned to ensure the removal of all the dye used. Therefore, initially, three interventions were performed in three models with Normal Middle Turbinate; then the middle turbinates of the three models were sutured, and the three interventions in the three models were performed again.

An image of each sinus was captured for each intervention in each MT group, totaling 144 images (1 image × 3 interventions × 2 MT groups × 4 sinuses × 6 sides), which were placed in a PowerPoint presentation and numbered 1 through 144, with no indication of the middle turbinate group or topical therapy intervention performed.

**Figure 3:**
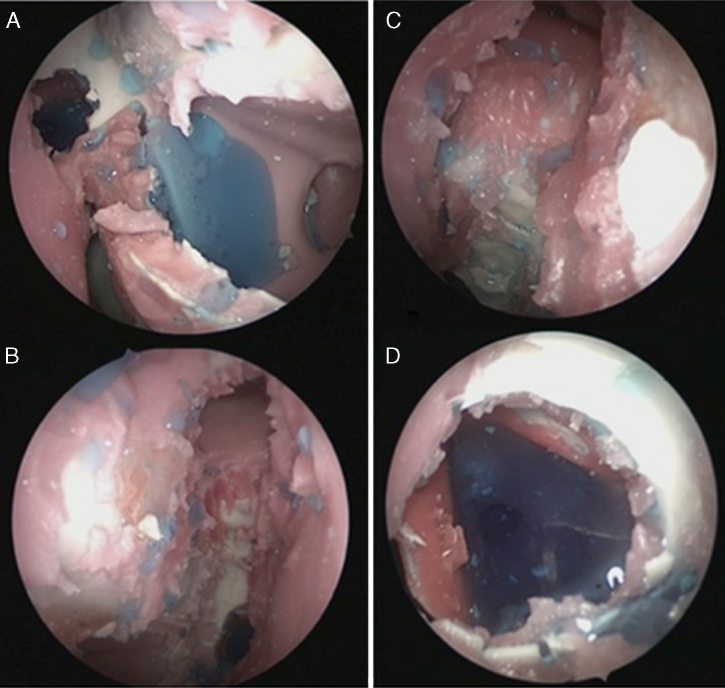
Examples of high amount of fluid in the sinuses. (A) Left maxillary sinus; (B) right Ethmoid sinus; (C) right frontal sinus; (D) left sphenoidal sinus.

The images were classified according to the amount of residual volume in a semi-quantitative scale. The scores that could be attributed to each sinus were: 0 (no fluid in the cavity), 1 (low amount of fluid in the cavity), 2 (moderate amount of fluid in the cavity) and 3 (large amount of fluid in the cavity). All images were analyzed by ten evaluators, who were trained otolaryngologists, who were not involved in topical therapies or image scanning, totaling 1440 analyzes. To standardize the assessment, the authors previously showed examples to the evaluators of what they considered a large amount of fluid in each of the sinuses (Fig. 3A–D). The scores 0 and 1 have been considered Poor Penetration to the sinuses, while the scores 2 and 3 were considered Good Penetration.

The statistical analysis consisted of the chi-square test (or Fisher's exact when necessary), considering categorical variables (Bad or Good Penetration) in the groups studied (Spray *vs.* Syringe *vs.* Squeeze, or Normal Middle Turbinate *vs.* Sutured Middle Turbinate). Inter-observer concordance analysis was assessed using the kappa test. For all statistical tests *p* values lower than 5% were considered significant.

## Results

Inter-observer agreement was considered large (Kappa = 0.628, *p* < 0.001; 0.604–0.653).

The three interventions showed to be different as for sinus penetration (*p* < 0.0001). Syringe intervention was more effective than the Spray one (68% of good reviews for the sinus penetration *vs.* 8%), but poorer than the Squeeze intervention (68% *vs.* 78%), as shown in Table 1.

**Table 1:** Penetration of topical therapy in each intervention.

Intervention	Penetration of topical therapy				Total
	Bad		Good		
	*n*	%	*n*	%	*n*
Spray	441	91.9%	39	8.1%	480
Syringe	152	31.7%	328	68.3%	480
Squeeze	104	21.7%	376	78.3%	480
Total	697	48.4%	743	51.6%	1440

*n*, number; %, percentage.

Chi-square test: *p* < 0.0001.

When considering the status of the middle turbinate, it was noted that when the middle turbinate was sutured, topical therapies showed higher penetration than when the middle turbinate was in normal position in the postoperative period, as shown in Table 2.

**Table 2:** Penetration of topical therapy according to the middle turbinate.

Middle turbinate	Penetration of topical therapy				Total
	Bad		Good		
	*n*	%	*n*	%	*n*
Normal	373	51.8%	347	48.2%	720
Sutured	324	45.0%	396	55.0%	720
Total	697	48.4%	743	51.6%	1440

*n*, number; %, percentage.

Chi-square test: *p* = 0.01.

Considering the penetration of topical therapies according to the status of the middle turbinate, it was noted that in the Normal Middle Turbinate case, the initial pattern of penetration, Squeeze > Syringe > Spray, was kept (Table 3). However, when considering only the Sutured Middle Turbinate case, the Syringe intervention proved to be similar to the Squeeze (76% *vs.* 80%, *p* = 0.27) (Table 4).

**Table 3:** Penetration of topical therapy with Normal Middle Turbinate.

Intervention	Penetration of topical therapy				Total
	Bad		Bad		
	*n*	%	*n*	%	*n*
Spray	221	92.1%	19	7.9%	240
Syringe	95	39.6%	145	60.4%	240
Squeeze	57	23.8%	183	76.3%	240
Total	373	51.8%	347	48.2%	720

*n*, number; %, percentage.

Chi-square Test: Spray *vs*. Syringe *vs*. Squeeze *p* < 0.0001; Spray *vs*. Syringe *p* < 0.0001; Spray *vs*. Squeeze *p* < 0.0001; Syringe *vs*. Squeeze *p* < 0.0001.

**Table 4:** Penetration of topical therapy with Sutured Middle Turbinate.

Intervention	Penetration of topical therapy				Total
	Bad		Good		
	*n*	%	*n*	%	*n*
Spray	220	91.7%	20	8.3%	240
Syringe	57	23.8%	183	76.3%	240
Squeeze	47	19.6%	193	80.4%	240
Total	324	45.0%	396	55.0%	720

*n*, number; %, percentage.

Chi-square test: Spray *vs*. Syringe *vs*. Squeeze *p* < 0.0001; Spray *vs*. Syringe *p* < 0.0001; Spray *vs*. Squeeze *p* < 0.0001; Syringe *vs*. Squeeze *p* = 0.27.

## Discussion

The present study demonstrated that topical treatment with 60-mL syringe was more effective than nasal spray regardless of the middle turbinate situation, and was as effective as the squeeze bottle intervention when administered following middle turbinate suturing. For the Brazilian reality, this study proved to be essential, since there are no squeeze bottles commercially available in this market, and the topical irrigation therapies are routinely performed with syringes. The only Brazilian study published to date with irrigation of corticosteroids was administered with 20-mL syringes, showing that this is actually the national reality.^19^

There are not many topical therapy studies of irrigation with syringes. Snidvongs et al.^20^ compared sinus penetration after topical therapy with 50-mL syringes (using 40 mL of solution) and nasal spray (10 mL). However, unlike the present study that evaluated the sinus penetration post-ESS, the study by Snidvongs et al. evaluated patients with CRS with no previous surgery, proving that the sinus penetration in non-operated sinuses is negligible, regardless of the technique used. Even so, the volume retained in the nasal cavity was significantly higher with the 50-mL syringe.^20^

Abadie et al.^10^ performed a study on cadavers, all after ESS, which compared various irrigation techniques, divided into high-volume irrigations, and sprays, where better results of sinus penetration results with high-volume irrigation was observed, similarly to this study. Of the several high-volume irrigation techniques, the squeeze bottle intervention was more effective, with this model being used in our study. The confirmation of this study that topical therapy with a syringe is more effective than the spray is enough to justify its routine use in our national reality in cases of failure of the spray.^19^ Moreover, the finding that the syringe is almost as efficacious as the squeeze bottle (considered to be the best technique of topical therapy in the study by Abadie et al.^10^) when the middle turbinate is sutured is essential to confirm that the use of the syringe may be regarded as the best therapeutic option for patients with CRS postoperatively in our reality.

In addition, it is important to emphasize the role of surgery in the topical nasal therapy. Harvey et al.^21^ demonstrated that the status of the sinus ostia influences sinus penetration ability of topical therapy. Using high-volume techniques, the authors demonstrated that operated sinuses had higher penetration than controls with no disease, which in turn had higher penetration than CRS patients without prior surgery. Currently, one of the mainstays of ESS is exactly that of creating open cavities, accessible to topical therapy.^22^ The concept is purely mechanical, in which there is the need for physical irrigation access to the sinuses. Therefore, the middle turbinate, with its central position in the nasal cavity, could play a role in the efficacy of topical therapy. This study confirmed that the position of the middle turbinate can influence sinus penetration, with the transeptal suture of middle turbinates facilitating sinus penetration of topical therapies in general, and also equaling the efficiency of 60-mL syringe to that of the squeeze bottle. Therefore, it is necessary to treat the middle turbinate in order to improve the efficacy of topical therapy of irrigation with a syringe.

## Conclusion

In models of surgical nasal dissection, topical therapy of irrigation with a 60-mL syringe was more effective than that with nasal spray. The status of the middle turbinate proved to be fundamental and influenced topical therapy. Irrigation with a syringe was as effective as that with a squeeze bottle when the middle turbinate was sutured to the nasal septum.

## Conflicts of interest

The authors declare no conflicts of interest.

## References

[1] Anselmo-Lima W.T., Sakano E., Tamashiro E., Nunes A.A., Fernandes A.M., Pereira E.A. (2015). Rhinosinusitis: evidence and experience: October 18 and 19, 2013 – São Paulo. Braz J Otorhinolaryngol.

[2] Fokkens W.J., Lund V.J., Mullol J., Bachert C., Alobid I., Baroody F. (2012). European position paper on Rhinosinusitis and Nasal Polyps 2012. Rhinol Suppl.

[3] Timperley D., Schlosser R.J., Harvey R.J. (2010). Chronic rhinosinusitis: an education and treatment model. Otolaryngol Head Neck Surg.

[4] Snidvongs K., Kalish L., Sacks R., Craig J.C., Harvey R.J. (2011). Topical steroid for chronic rhinosinusitis without polyps. Cochrane Database Syst Rev.

[5] Kalish L., Snidvongs K., Sivasubramariam R., Cope D., Harvey R.J. (2012). Topical steroids for nasal polyps. Cochrane Database Syst Rev.

[6] Burns J. (1992). Nasal lavage. J Otolaryngol.

[7] Wingrave W. (1902). A clinical lecture on the nature of discharges and douches. Lancet.

[8] Harvey R., Hannan S.A., Badia L., Scadding G. (2007). Nasal saline irrigations for the symptoms of chronic rhinosinusitis. Cochrane Database Syst Rev.

[9] Wormald P.-J., Cain T., Oates L., Hawke L., Wong I. (2004). A comparative study of three methods of nasal irrigation. Laryngoscope.

[10] Abadie W.M., McMains K.C., Weitzel E.K. (2011). Irrigation penetration of nasal delivery systems: a cadaver study. Int Forum Allergy Rhinol.

[11] Harvey R.J., Schlosser R.J. (2009). Local drug delivery. Otolaryngol Clin North Am.

[12] Harvey R.J., Goddard J.C., Wise S.K., Schlosser R.J. (2008). Effects of endoscopic sinus surgery and delivery device on cadaver sinus irrigation. Otolaryngol Head Neck Surg.

[13] Friedman M., Landsberg R., Tanyeri H. (2000). Middle turbinate medialization and preservation in endoscopic sinus surgery. Otolaryngol Head Neck Surg.

[14] Bhalla R.K., Kaushik V., de Carpentier J. (2005). Conchopexy suture to prevent middle turbinate lateralisation and septal haematoma after endoscopic sinus surgery. Rhinology.

[15] Yanagisawa E., Joe J.K. (1999). The use of spacers to prevent postoperative middle meatal adhesions. Ear Nose Throat J.

[16] Soler Z.M., Hwang P.H., Mace J., Smith T.L. (2010). Outcomes after middle turbinate resection: revisiting a controversial topic. Laryngoscope.

[17] Shih C., Chin G., Rice D.H. (2003). Middle turbinate resection: impact on outcomes in endoscopic sinus surgery. Ear Nose Throat J.

[18] Biedlingmaier J.F. (1993). Endoscopic sinus surgery with middle turbinate resection: results and complications. Ear Nose Throat J.

[19] Kosugi E.M., Moussalem G.F., Simões J.C., de Souza R.P., Chen V.G., Saraceni-Neto P. (2016). Topical therapy with high-volume budesonide nasal irrigations in difficult-to-treat chronic rhinosinusitis. Braz J Otorhinolaryngol.

[20] Snidvongs K., Chaowanapanja P., Aeumjaturapat S., Chusakul S., Praweswararat P. (2008). Does nasal irrigation enter paranasal sinuses in chronic rhinosinusitis. Am J Rhinol.

[21] Harvey R.J., Debnath N., Srubiski A., Bleier B., Schlosser R.J. (2009). Fluid residuals and drug exposure in nasal irrigation. Otolaryngol Head Neck Surg.

[22] Rudmik L., Hoy M., Schlosser R.J., Harvey R.J., Welch K.C., Lund V. (2013). Topical therapies in the management of chronic rhinosinusitis – an evidence-based review with recommendations. Int Forum Allergy Rhinol.

